# Worldwide productivity and research trends of publications concerning stent application in acutely ruptured intracranial aneurysms: A bibliometric study

**DOI:** 10.3389/fneur.2022.1029613

**Published:** 2022-11-11

**Authors:** Rundong Chen, Yanpeng Wei, Guanghao Zhang, Renkun Zhang, Xiaoxi Zhang, Dongwei Dai, Qiang Li, Rui Zhao, Yi Xu, Qinghai Huang, Pengfei Yang, Qiao Zuo, Jianmin Liu

**Affiliations:** Neurovascular Center, Changhai Hospital, Naval Medical University, Shanghai, China

**Keywords:** stent application, stent-assisted coiling, ruptured intracranial aneurysms, bibliometric, cluster analysis, Citespace

## Abstract

**Background:**

Stenting is a common clinical practice to treat acutely ruptured intracranial aneurysm (RIA). Although multiple studies have demonstrated its long-term safety and effectiveness, there is currently a lack of bibliometric analysis on stent application in acutely RIA. This study sought to summarize the current status of research in this field and lay a foundation for further study.

**Materials and methods:**

Related publications were searched in the Web of Science Core Collection (WoSCC) database. Data analysis and visualization were performed by R and CiteSpace software.

**Results:**

A total of 275 publications published in English from 1997 to 2022 were included in this study. The growth of publications slowed down. The reference co-citation network identified 13 clusters with a significant network (Q = 0.7692) and convincing clustering (S = 0.9082). The research focus was acutely RIA and the application of stents during interventional procedures. The main trends of research were: (1) development of materials, and (2) safety of stent application in acutely RIA. The United States contributed the most articles, and Jianmin Liu was the most prolific author. Mayo Clinic was the leading institution in this field. Most articles were published in Interventional Neuroradiology.

**Conclusions:**

This study analyzed the research trends, hotspots and frontiers of stent application in acutely RIA. It is our hope that the results obtained could provide useful information to researchers to get a clearer picture about their future research directions in this field.

## Introduction

The past decades have witnessed remarkable advances in the endovascular treatment of acutely ruptured intracranial aneurysms (RIA), and the safety and effectiveness of stent application in acutely RIA have been explored (1). RIA is the most common cause of subarachnoid hemorrhage (SAH), which is often a devastating event with high mortality and morbidity (2). About 4% and 1% SAH patients have an increased risk of rebleeding in the first 24 h and every day in the first month respectively (3). Endovascular and surgical treatments are available for aneurysm repair, which are the only effective treatments to prevent rebleeding at present (4). However, for some complex aneurysms, giant aneurysms and aneurysms with a low fundus-to-neck ratio, specialized skills are required to obtain satisfactory embolization, including stent-assisted coiling (SAC), balloon-assisted coiling (BAC), flow diverters (FD), and the use of new embolic materials including liquids (5–7). Stent placement has been commonly applied in acutely RIA, including classic laser-cut stents, braided stents, drug-eluting stents, and covered stents (8–10). These skills are expected to enable aneurysms previously considered unsuitable for the endovascular procedure to be treated in the future (5, 11). Early studies suggested a high incidence of adverse events with stent application in acutely RIA, including stent-related thrombosis and hemorrhagic complications due to the use of antiplatelet drugs (12–14). However, with the progress in endovascular skills, materials, devices and antiplatelet strategies, the perioperative safety of stent application in acutely RIA has been continuously improved (1, 15–17). Exploration and summary of the research trends of stent application in acutely RIA treatment is significant to those who want to carry out this research.

Bibliometrics uses statistical methods to analyze publications, especially those of scientific content. Bibliometric mapping allows data to be presented in ways that make relationships more understandable and provide researchers with relatively macro information (18). The method of bibliometric analysis has become increasingly mature and has been widely used in clinical disease research (19).

However, there is a lack of data on bibliometric analysis of stent application in acutely RIA. To fill this gap, we conducted a bibliometric study to discuss publications on stent application in acutely RIA from 1997 to 2022 both quantitatively and qualitatively. In addition, we summarized the main research trends and frontiers, provided the latest insights and findings, and looked forward to the future development of this field.

## Materials and methods

### Objectives

The primary objective of the study was to systematically map how stenting is evolved in the treatment of patients with acutely RIA, and identify the main trends and hot topics of research in this field by constructing networks of co-cited references and co-occurring keywords. The secondary objective was to render the research network in terms of countries, authors, institutions and journals.

### Data collection

We searched publications from the Web of Science Core Collection (WoSCC) through the Science Citation Index Expanded (SCI-E). The search terms combined Medical Subject Headings words and keywords:[TS = (stent^*^) OR TS=(“stent assisted”)] AND TS=(“ruptured intracranial aneurysm^*^”). The language was limited to “English.” The main document type was “articles” and “reviews” with no time limitation. All the search result records, including the title, author information, keyword, abstract and reference were exported in TXT format for analysis on July 15, 2022.

### Data analysis

The raw files were analyzed by R software (4.1.3) and Citespace software (6.1.R2). The “bibliometrix” R package is an open-source tool for quantitative research in bibliometrics. It summarizes the preliminary information, country scientific production, and the cumulate occurrence of journal articles in this study. CiteSpace is a Java application for visualizing patterns and trends in scientific publications by focusing on identifying critical points in developing a particular field. It was used to explore networks of co-cited references and co-occurring keywords, as well as collaboration networks between countries, authors, institutions, and journals.

## Results

### General overview

**Figure 1:**
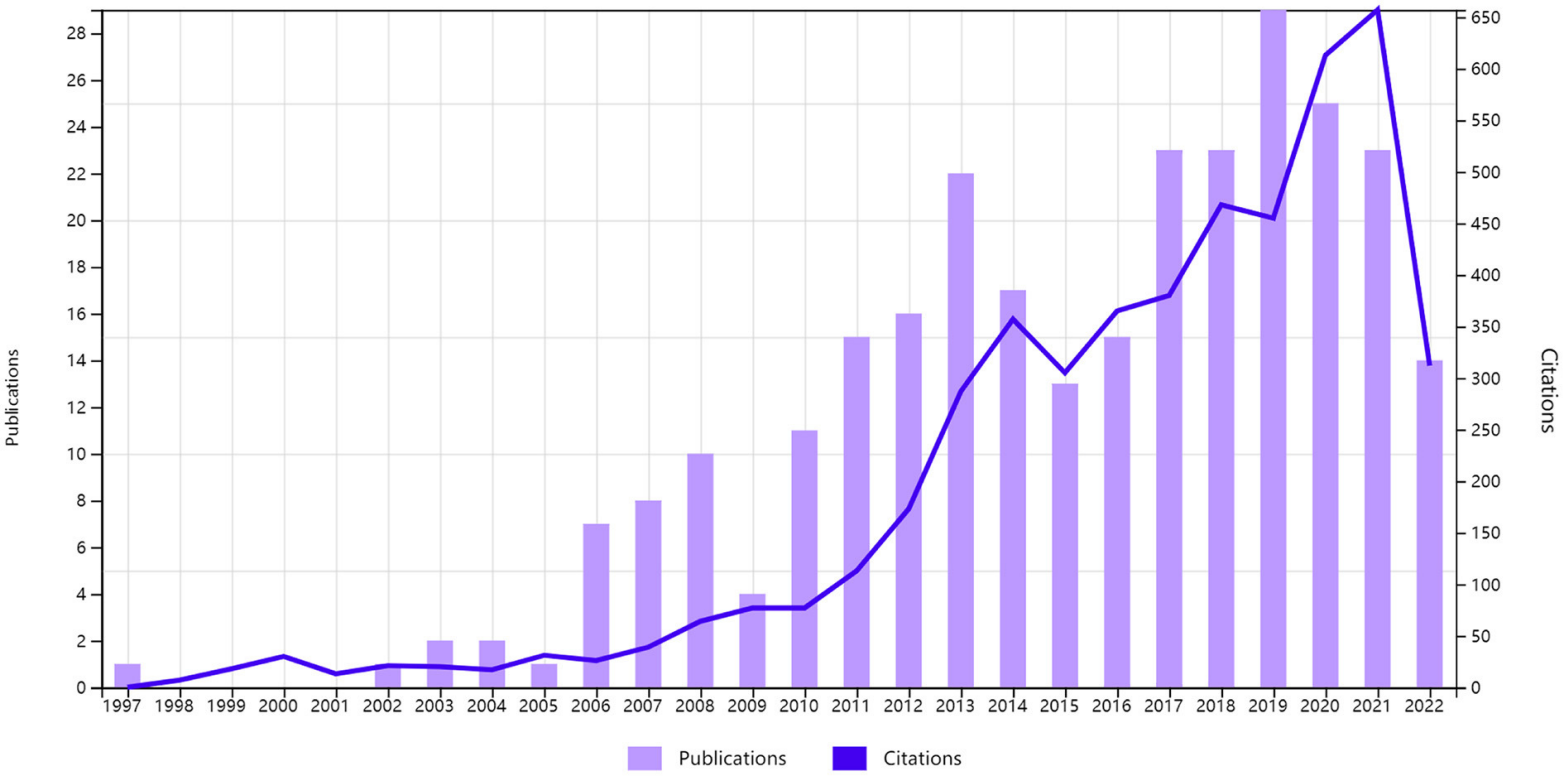
Annual scientific production and citation.

A total of 275 publications about stent application in acutely RIA from 1997 to 2022 were included in this study, of which 236 were original articles, and 39 were review articles (Figure 1). The growth of the overall number of articles and the mean article citations per year slowed down. The COVID-19 pandemic may lead to a decline. The cumulative number of citations for these publications was 5,041 (4,464 without self-citations), with a mean number of citations per item of 18.33. The mean H-index in this field was 38. The analysis showed significant progress in this field in the past 20 years, especially in 2013 and 2019.

### Co-cited references and references

**Figure 2:**
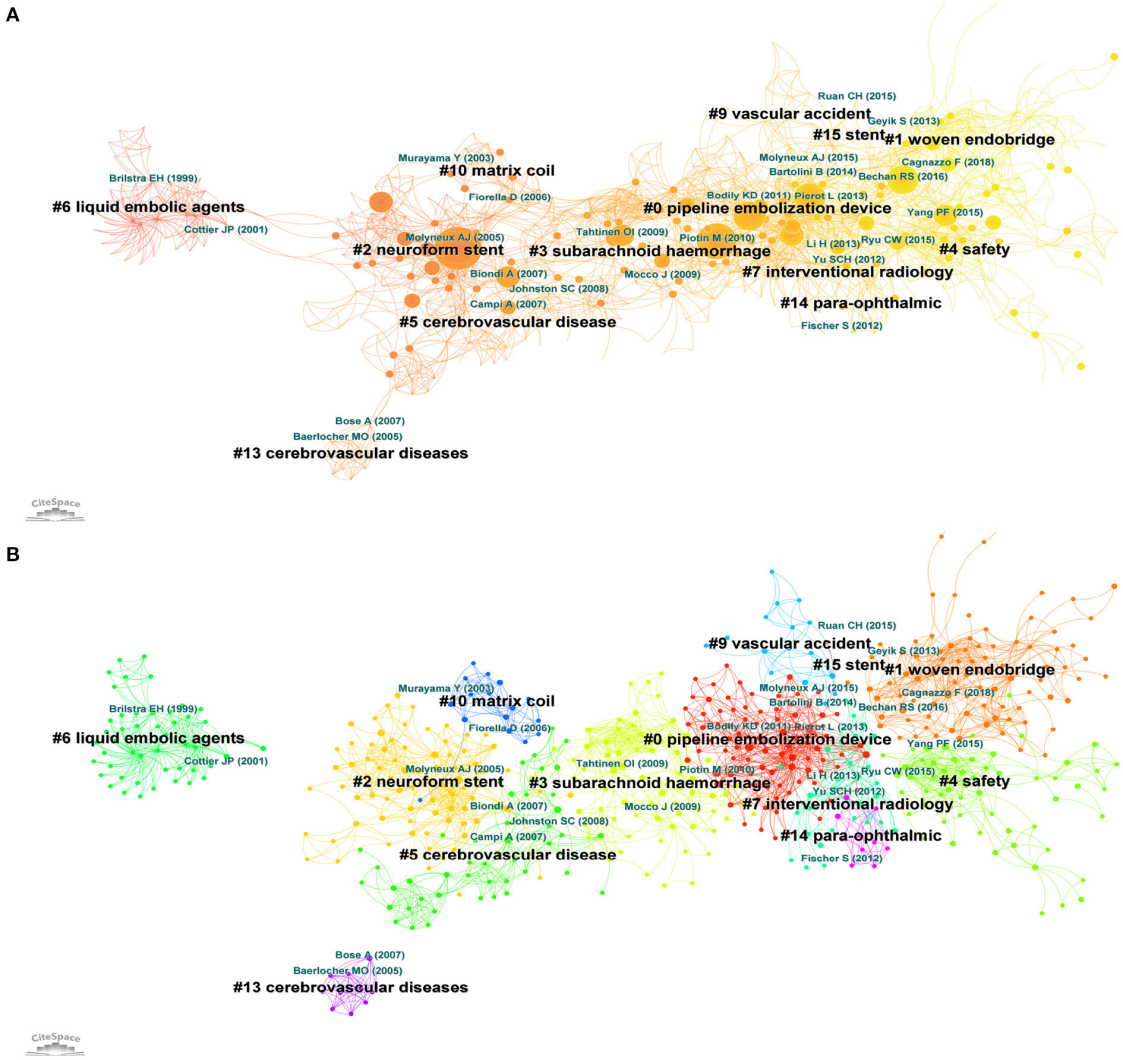
**(A)** Co-citation reference network with cluster visualization. The node's size (article) is proportional to the number of times the publication has been co-cited. **(B)** Visualization map of the corresponding clusters. Publication topics of the same type are clustered in the same color block.

Co-citation references were two or more articles appearing simultaneously in the references of other documents. The association of co-citations may reveal how groupings have evolved independently from the original publication (19). The top 10 most cited documents and the top 10 most cited references were shown in Tables 1, 2 respectively. There were 693 nodes and 2,830 links constructed by Citespace for a map of reference co-citations with corresponding clusters (Figures 2A,B). The first article was issued in 1997 (20). At that time, stenting was attempted for the treatment of acutely RIA through endovascular therapy. Thirteen clusters were identified in this network with significant modularity Q scores and silhouette scores (Q = 0.7692, S = 0.9082). We found a research focus and two different research trends in this map. The research focus was acutely SAH and the application of stents during interventional procedures. These clusters, with the indication of the label, silhouette score, size, the mean year of publications, and most representative reference were: cluster#5 (cerebrovascular disease, S = 0.912, size = 49, mean year = 2009) (21), cluster#3 (subarachnoid hemorrhage, S = 0.903, size = 71, mean year = 2011) (22), cluster #7 (interventional radiology, S=0.864, size = 32, mean year = 2016) (23), and cluster#15 (stent, S = 0.994, size = 5, mean year = 2018) (24). The first trend was concerned with the development of materials. It started with cluster #6 (liquid embolic agents, S = 0.993, size = 48, mean year = 2002) (25), which developed research on cluster #2 (neuroform stent, S = 0.908, size = 81, mean year = 2007) (26) and cluster #10 (matrix coil, S = 0.959, size = 16, mean year = 2008) (27). More recently, these clusters became cluster #0 (pipeline embolization device, S = 0.851, size = 92, mean year = 2014) (28), with strong links to cluster#1 (woven endobridge, S = 0.875, size = 89, mean year = 2020) (29). The second major research trend was concerned with the safety of stent application in acutely RIA. This trend began with cluster #9 (vascular accident, S = 0.952, size = 17, mean year = 2014) (30) and cluster #14 (para-ophthalmic, S = 0.996, size = 10, mean year = 2016) (31), which has currently evolved into cluster#4 (safety, S = 0.941, size = 59, mean year = 2019) (32).

**Table 1:** The top 10 most cited documents.

**Local** **citations^a^**	**Global** **citations^b^**	**Year**	**Source**	**Title**	**Doi**
43	171	2011	American Journal of Neuroradiology	Stent-assisted coiling in acutely ruptured intracranial aneurysms: a qualitative, systematic review of the literature	10.3174/ajnr.A2478
27	75	2012	Neurosurgery	Stent-assisted coiling of wide-necked aneurysms in the setting of acute subarachnoid hemorrhage: experience in 65 patients	10.1227/NEU.0b013e318246a4b1
23	62	2015	American Journal of Neuroradiology	Complications in Stent-Assisted Endovascular Therapy of Ruptured Intracranial Aneurysms and Relevance to Antiplatelet Administration: A Systematic Review	10.3174/ajnr.A4365
18	57	2014	Journal of Neurosurgery	Stent-assisted coil embolization of ruptured wide-necked aneurysms in the acute period: incidence of and risk factors for periprocedural complications	10.3171/2014.4.JNS131662
16	85	2015	Journal of NeuroInterventional Surgery	Utilization of Pipeline embolization device for treatment of ruptured intracranial aneurysms: US multicenter experience	10.1136/neurintsurg-2014-011320
15	31	2012	Journal of NeuroInterventional Surgery	Stent assisted coiling of the ruptured wide necked intracranial aneurysm	10.1136/neurintsurg-2011-010035
14	344	1997	Journal of NeuroInterventional Surgery	Intravascular stent and endovascular coil placement for a ruptured fusiform aneurysm of the basilar artery. Case report and review of the literature	10.3171/jns.1997.87.6.0944
14	95	2012	American Journal of Neuroradiology	Immediate and midterm results following treatment of recently ruptured intracranial aneurysms with the Pipeline embolization device	10.3174/ajnr.A2797
13	99	2011	Neurosurgery	Stent-associated flow remodeling causes further occlusion of incompletely coiled aneurysms	10.1227/NEU.0b013e3182181c2b
11	99	2012	Neurosurgery	Safety and efficacy of endovascular treatment of basilar tip aneurysms by coiling with and without stent assistance: a review of 235 cases	10.1227/NEU.0b013e318265a416

a Number of citations in the network of 275 literature.

b Number of citations in the literature according to the journal where the paper was published.

**Table 2:** The top 10 most cited references.

**Local** **citations**	**Global** **citations**	**Year**	**Source**	**Title**	**Doi**
127	2,444	2002	Lancet	International Subarachnoid Aneurysm Trial (ISAT) of neurosurgical clipping vs. endovascular coiling in 2,143 patients with ruptured intracranial aneurysms: a randomized trial	10.1016/s0140-6736(02)11314-6
105	1,518	2005	Lancet	International subarachnoid aneurysm trial (ISAT) of neurosurgical clipping vs. endovascular coiling in 2,143 patients with ruptured intracranial aneurysms: a randomized comparison of effects on survival, dependency, seizures, rebleeding, subgroups, and aneurysm occlusion	10.1016/S0140-6736(05)67214-5
68	1,061	2003	Stroke	Long-term angiographic recurrences after selective endovascular treatment of aneurysms with detachable coils	10.1161/01.STR.0000073841.88563.E9
43	171	2011	American Journal of Neuroradiology	Stent-assisted coiling in acutely ruptured intracranial aneurysms: a qualitative, systematic review of the literature	10.3174/AJNR.A2478
43	444	2010	Stroke	Stent-assisted coiling of intracranial aneurysms: clinical and angiographic results in 216 consecutive aneurysms	10.1161/STROKEAHA.109.558114
43	2,380	2003	Lancet	Unruptured intracranial aneurysms: natural history, clinical outcome, and risks of surgical and endovascular treatment	10.1016/S0140-6736(03)13860-3
39	596	2003	Journal of Neurosurgery	Guglielmi detachable coil embolization of cerebral aneurysms: 11 years' experience	10.3171/JNS.2003.98.5.0959
33	298	2004	Neurosurgery	Endovascular occlusion of wide-necked aneurysms with a new intracranial microstent (Neuroform) and detachable coils	10.1227/01.NEU.0000124484.87635.CD
33	246	2013	Stroke	Stent-assisted coiling of intracranial aneurysms: predictors of complications, recanalization, and outcome in 508 cases	10.1161/STROKEAHA.111.000641
31	121	2009	Radiology	Wide-necked intracranial aneurysms: treatment with stent-assisted coil embolization during acute (<72 h) subarachnoid hemorrhage–experience in 61 consecutive patients	10.1148/RADIOL.2531081923

### Keywords and hotspots

**Figure 3:**
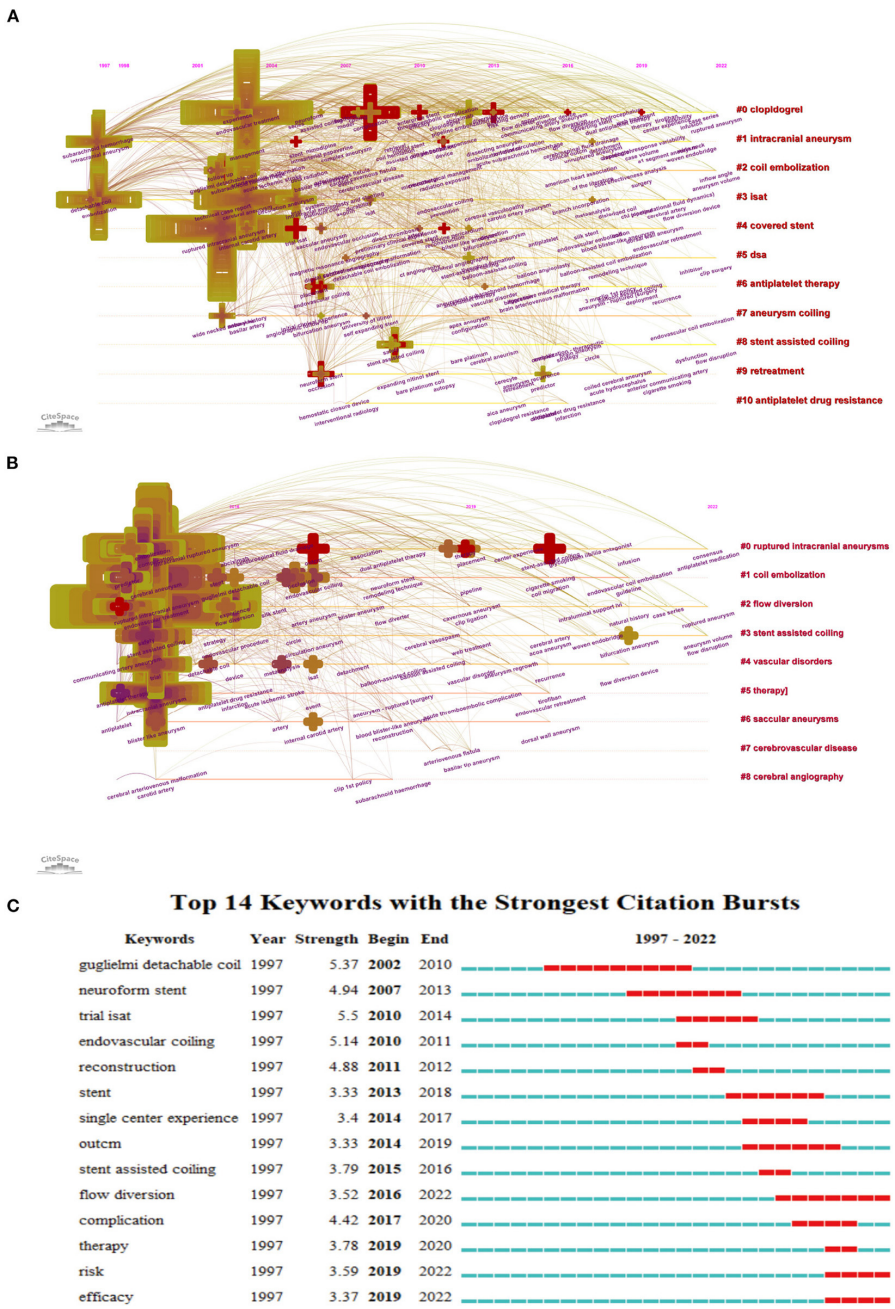
Timeline visualization of co-occurring author keyword networks [**(A)** 1980–2021 and **(B)** 2015–2022]. The size of a cross is proportional to the burstness of keywords co-occurrence. The co-occurring keyword network is weighted on total link strength across different keyword nodes and scored on the mean publication years. The clusters are labeled in red at the right of the timeline maps. **(C)** Top14 keywords with the strongest citation bursts.

We extracted the timeline of the co-occurring keywords network (1997–2022) by Citespace (Figure 3A). Eleven clusters of keywords were identified with modularity Q score = 0.3962 and silhouette score = 0.7111. The most critical cluster was “clopidogrel,” followed by “intracranial aneurysm,” “coil embolization,” “isat” (international subarachnoid aneurysm trial), “covered stent,” “dsa,” “antiplatelet therapy,” “aneurysm coiling,” “stent assisted coiling,” “retreatment,” and “antiplatelet drug resistance.” We further extracted the same network from 2015 to 2022 (Figure 3B), and identified nine clusters of keywords with modularity Q score = 0.3924 and silhouette score = 0.7414. The most essential cluster was “ruptured intracranial aneurysm,” followed by “coil embolization,” “flow diversion,” “stent assisted coiling,” “vascular disorders,” “therapy,” “endovascular occlusion,” “cerebrovascular disease,” and “neuroradiography.” Moreover, keyword bursts represented keywords that were frequently cited over a period of time (Figure 3C). The earliest burst keyword was “Gugliemi detachable coil,” which began in 2002 and lasted 8 years. Subsequently emerging keywords were “neuroform stent,” “trial isat,” “endovascular coiling,” and “reconstruction,” all of which focused on the feasibility of the stent application in acutely RIA. These keywords further evolved into “stent,” “single center experience,” “outcome,” and “stent assisted coiling” in 2013, which were mainly concerned with the safety of stent application in acutely RIA. More recently, these keywords became “flow diversion,” “complication,” “therapy,” “risk,” and “efficacy.”

### Countries and regions

**Figure 4:**
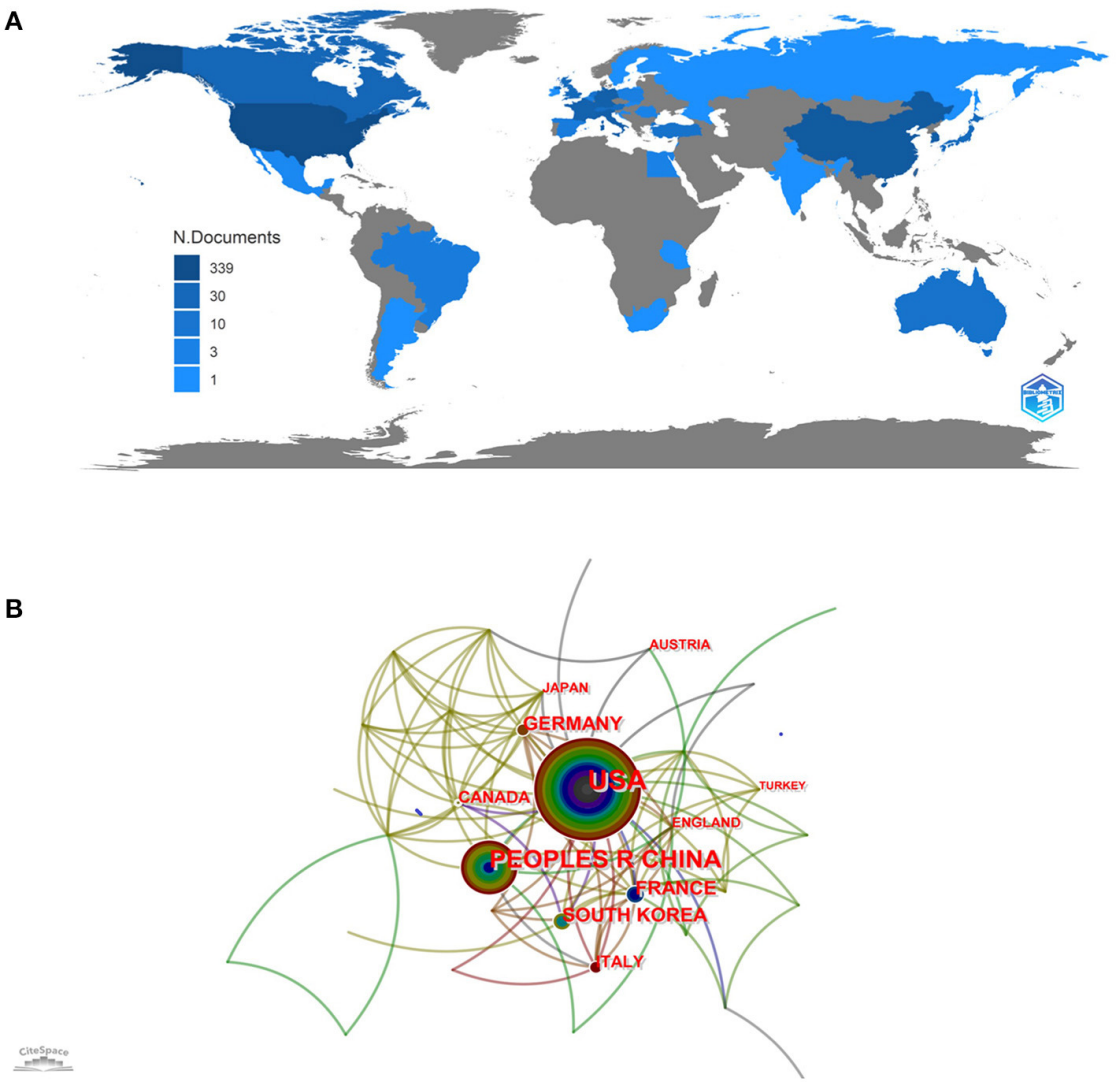
**(A)** Country-specific production. Dark blue = high productivity. Gray = no documents. **(B)** Country cooperation network map. The size of each node represents the number of publications published by the country, and the thickness of each link represents the strength of the cooperative relationship between two countries.

Based on the analysis of cooperation networks across countries or regions, 37 countries or regions were identified, of which the United States (US) contributed the most with 102 publications, followed by the People's Republic of China (*n* = 57), South Korea (*n* = 29), Germany (*n* = 22), and France (*n* = 21) (Supplementary Table 1). A country scientific production map was shown in Figure 4A and the cooperation networks across countries were mapped in Figure 4B. The US, as the landmark node, had extensive collaborations with other countries or regions worldwide.

### Authors and institutions

**Figure 5:**
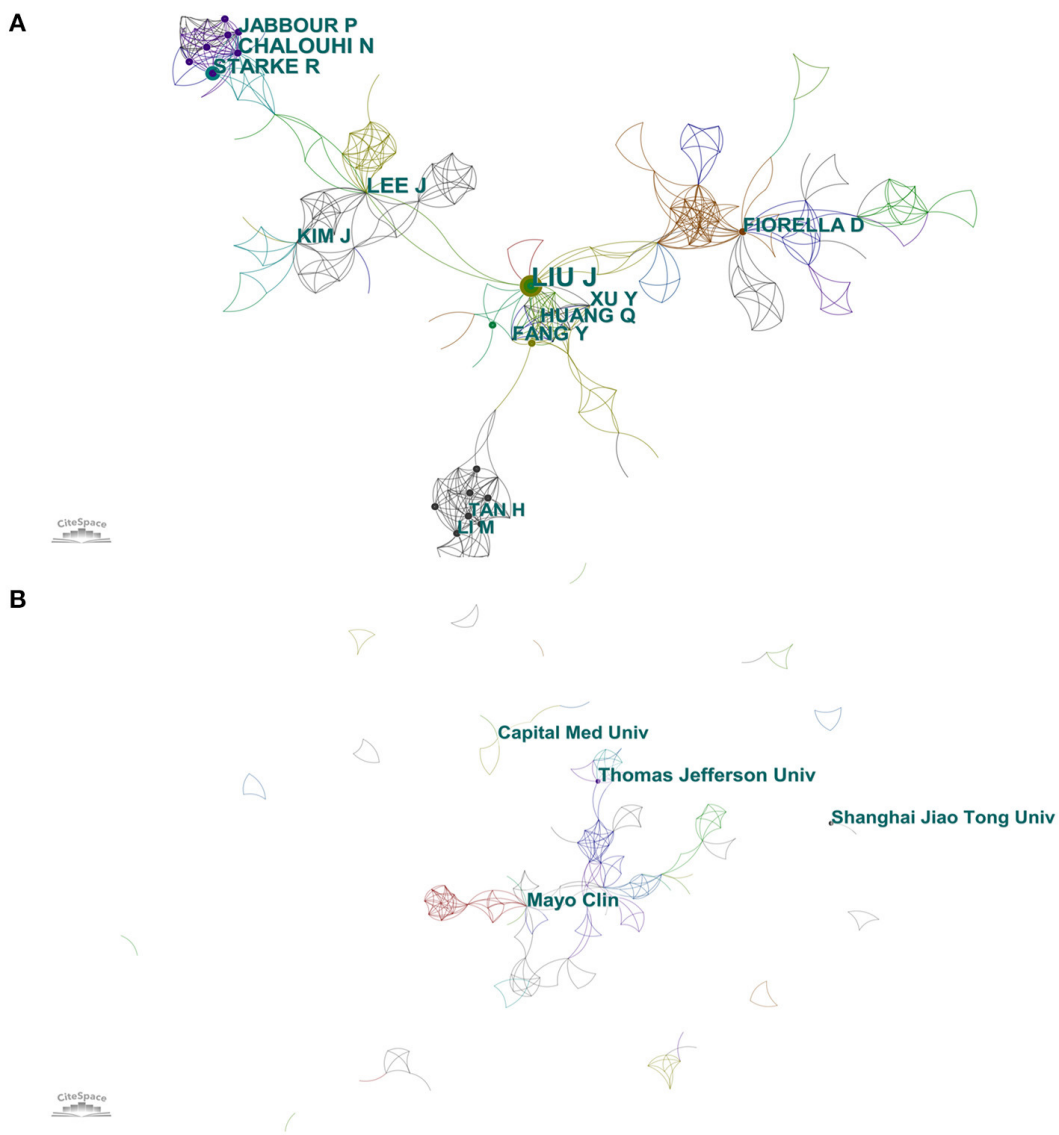
**(A)** Author cooperation network map. **(B)** Institution cooperation network map.

The cooperation network between authors is shown in Figure 5A, and the top 10 influential authors are shown in Supplementary Table 2. There were 477 nodes and 1,075 links, and the results showed that Jianmin Liu contributed the largest number (*n* = 16) of publications with the highest centrality (0.17), followed by Jeongjun Lee (*n* = 9), NohraChalouhi (*n* = 9), Robert M Starke (*n* = 8), and David J Fiorella (*n* = 7). Jianmin Liu and Jeongjun Lee constituted the two pivot nodes that connected the network diagram, which was why these two authors had a higher degree of centrality. The collaboration between other authors was relatively decentralized. The cooperation network between institutions is shown in Figure 5B. The top 5 institutions by citation counts were Mayo Clinic (*n* = 9), Thomas Jefferson University (*n* = 9), Shanghai Jiao Tong University (*n* = 8), Capital Medical University (*n* = 8), and Jefferson Hospital for Neuroscience (*n* = 7) (Supplementary Table 3).

### Journals

**Figure 6:**
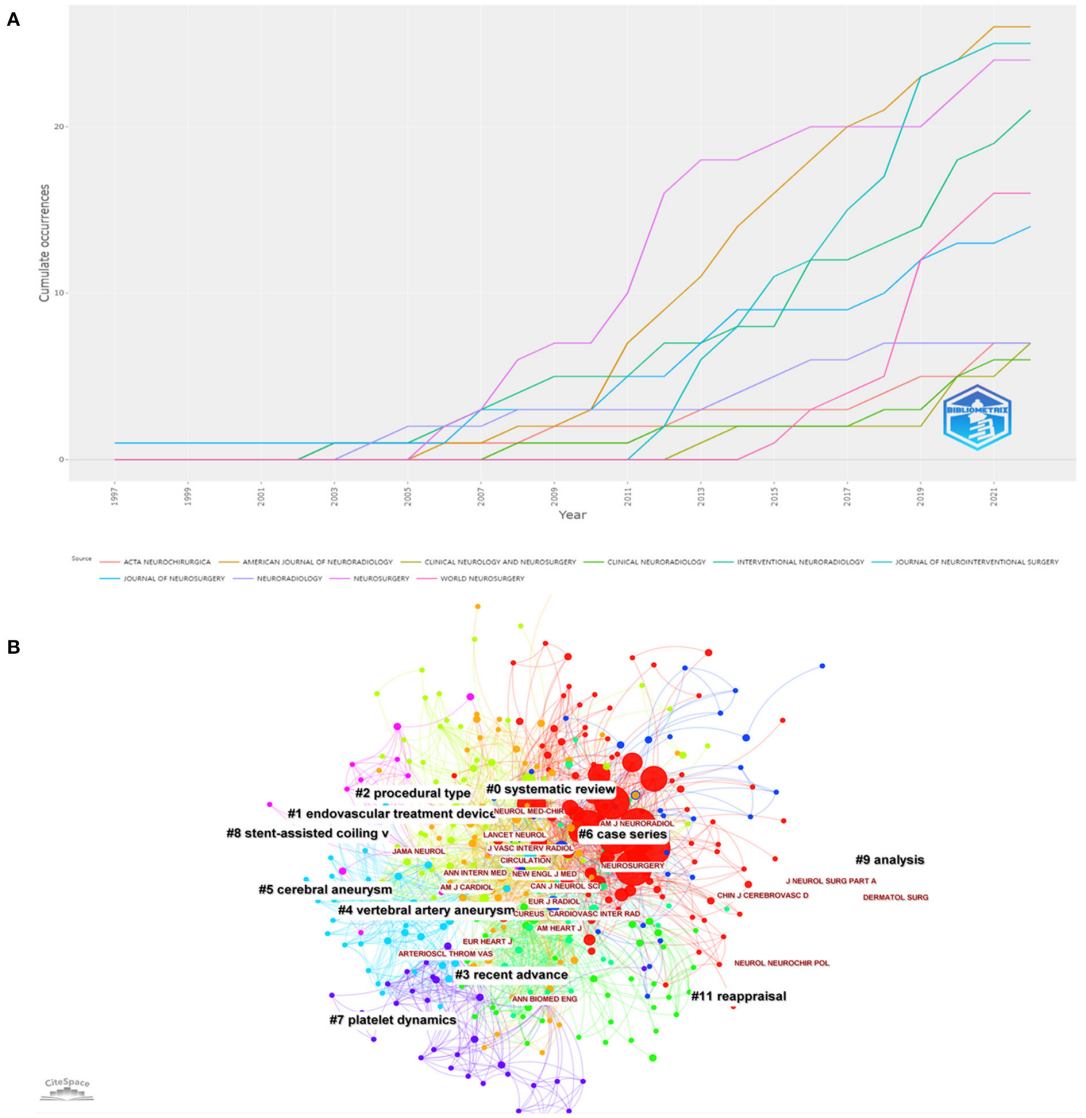
**(A)** Source growth of publications. **(B)** The co-cited journal network with cluster visualization.

The top five journals with the most references were the American Journal of Neuroradiology (*n* = 26), Journal of NeuroInterventional Surgery (*n* = 25), Neurosurgery (*n* = 24), interventional Neuroradiology (*n* = 21), and World Neurosurgery (*n* = 16) (Figure 6A, Supplementary Table 4). The co-cited journal network over the past 20 years is shown in (Figure 6B). The American Journal of Neuroradiology, Neurosurgery, Journal of Neurosurgery, Stroke and Lancet were the top five journals that enjoyed the largest number of citations (Supplementary Table 5). This shows that the American Journal of Neuroradiology made the most outstanding contribution and had the greatest influence in this field.

## Discussion

This study revealed the overall research results of stent application in acutely RIA in the past 25 years. The annual number of papers and literature trends may reflect the development speed and progress of research in this field. Before 2006, the number of publications in this field was roughly the same yearly. From 2006 to 2019, the number of publications increased obviously, reflecting the growing interest in this field, especially the evolution of materials and the safety in acutely RIA.

The co-cited references with the corresponding cluster network (1997–2022) described the coherent links between 13 different clusters and revealed the evolution of research trends about stent application in acutely RIA. The first trend was the evolution of materials, from liquid embolic agents to neuroform stents, then pipeline embolization devices and woven endobridge (WEB). The development of these new and exciting devices and materials has helped neurointerventionalists successfully treat aneurysmal SAH (16, 33, 34). But as the surface of the current stents is highly thrombotic, a dual antiplatelet regimen is required, which is still a controversial issue in the acute stage of SAH (35, 36). Therefore, the second major trend is the safety of stent application research in acutely RIA. In this aspect, the most concerned issues of researchers are the risk of increased hemorrhagic complications and acute stent thrombosis or even thromboembolic occlusion (37, 38), especially the ophthalmic artery (39). In addition, the focus on the past seven years showed that the latest research trends also aimed at developing materials and exploring technical security (Supplementary Figure 1). Analysis of the co-occurring keyword networks and burst keywords such as clopidogrel, ISAT, covered stent and antiplatelet resistance also verified these findings. The burst keywords in recent years, including flow diversion, complications, therapy, risk, and efficacy, also confirmed the current hot topic and research focus (Supplementary Figure 2). The safety and efficacy of stent application in acutely RIA have been a hot topic of discussion in recent years (32, 40, 41). Antiplatelet strategies have been found to be closely related to increased hemorrhagic complications. Furthermore, early use of anticoagulants after stent application in acutely RIA was identified as a risk factor for postoperative hemorrhagic complications. And dual antiplatelet agents were preferred by DELPHI consensus as a standard approach with aspirin and a glycoprotein IIb/IIIa receptor (35). Alternations based on anti-thrombogenic device coating might make stents used safely in the treatment of RIA (42). In terms of material selection, stents and coils made from LVIS and hydrogel are safer in the treatment of ruptured aneurysms because they can provide a higher immediate embolization rate (43, 44). For blood blister-like aneurysms, FD was a more sensible choice at present, owing to its high metal coverage and the change in blood flow to promote intrasaccular thrombosis for better isolating blood from entering the aneurysm (45). Although a wealth of experience and treatment guidelines have been gleaned from 25 years of research into stent application in acutely ruptured intracranial aneurysms, many questions still need to be further addressed. Future research may focus on the development of novel stent materials which would reduce the reliance on antiplatelet drugs during the perioperative period and consequently reduce the potential risk of hemorrhagic complications.

As shown in Supplementary Table 1, the country with the most significant number of publications was the US. Centrality represents the algorithm that calculates unweighted shortest paths between all pairs of nodes in a graph. The US had the highest centrality of 0.87, indicating that the US occupied a leading position in this field. The further cluster analysis revealed that the US and the People's Republic of China mainly focused on preoperative evaluation of stent application in acutely RIA (46) (Supplementary Figure 3). The most productive author was Jianmin Liu, and the author team headed by him was mainly concerned with stent placement (1). Studies by other authors were more concerned with endobridge devices and balloon remodeling (47) (Supplementary Figure 4). Among the top ten research institutions, five were in the US, four were in the People's Republic of China, and the rest in South Korea. However, the centrality of institutions is low, indicating a lack of academic collaboration between institutions. Nevertheless, cluster analysis suggested that some institutional collaborations still contributed to the multicenter experience and perioperative preparation of stent application in acutely RIA (48) (Supplementary Figure 5). Publication source analysis can help researchers identify core journals in their fields, and top-ranked co-cited journals can serve as authoritative references. The American Journal of Neuroradiology had the greatest number of articles published and the greatest number of articles cited simultaneously. In addition, those top co-cited and prolific journals mainly published systematic reviews, endovascular treatment devices, and recent advances in this field (29, 49) (Supplementary Figure 6).

This study inevitably had some limitations. The data were simply retrieved from the WoSCC database, which may lead to incomplete literature collection. In addition, the literature retrieved was limited to articles published in English, leading to some linguistic bias in the study results. With further research and exploration in this field, the findings of this study may be different from the realistic results in the future.

## Conclusion

To the best of our knowledge, this is the first systematic and multidimensional analysis of the research trends, hotspots and frontiers of stent application in acutely RIA in an objective way, which we hope can be used as a comprehensive guide for clinicians and scholars engaged in this field, and help researchers get a clearer picture of their future research directions.

## Data availability statement

The original contributions presented in the study are included in the article/Supplementary material, further inquiries can be directed to the corresponding authors.

## Author contributions

RC, YW, and GZ made substantial contributions to the conception and design, acquisition of data, analysis, and drafting of the manuscript. QZ, JL, RZhang, XZ, DD, QL, RZhao, YX, QH, and PY assisted in the evaluation of analysis and their interpretation. All authors read and approved the final manuscript.

## Funding

This research was supported by the 234 Discipline Peak Climbing Program of Changhai Hospital (2020YXK060).

## Conflict of interest

The authors declare that the research was conducted in the absence of any commercial or financial relationships that could be construed as a potential conflict of interest.

## Publisher's note

All claims expressed in this article are solely those of the authors and do not necessarily represent those of their affiliated organizations, or those of the publisher, the editors and the reviewers. Any product that may be evaluated in this article, or claim that may be made by its manufacturer, is not guaranteed or endorsed by the publisher.

## Supplementary material

Supplementary Figure 1 **(A)** Co-citation reference network with cluster visualization from 2015 to 2022. **(B)** Visualization map of the corresponding clusters from 2015 to 2022. **(C)** Timeline visualization of co-citation references network. **(D)** Top 12 references with the strongest citation bursts.

Supplementary Figure 2 Co-occurring author keyword networks with cluster visualization [**(A)** 1980–2021 and **(C)** 2015–2022]. Visualization map of the corresponding clusters [**(B)** 1980–2021 and **(D)** 2015–2022].

Supplementary Figure 3 **(A)** Country cooperation network with cluster visualization. **(B)** Timeline visualization of the country cooperation network.

Supplementary Figure 4 **(A)** Author cooperation network with cluster visualization. **(B)** Timeline visualization of the author cooperation network.

Supplementary Figure 5 **(A)** Institution cooperation network with cluster visualization. **(B)** Timeline visualization of the institution cooperation network.

Supplementary Figure 6 **(A)** Co-cited journal network map. **(B)** Timeline visualization of the co-cited journals network map.
