# Supplementary figures and images for: Worldwide productivity and research trends of publications concerning stent application in acutely ruptured intracranial aneurysms: A bibliometric study
Source: Front Neurol. 2022 Nov 11;13:1029613. doi: 10.3389/fneur.2022.1029613 (PMC9694826; doi:10.3389/fneur.2022.1029613)

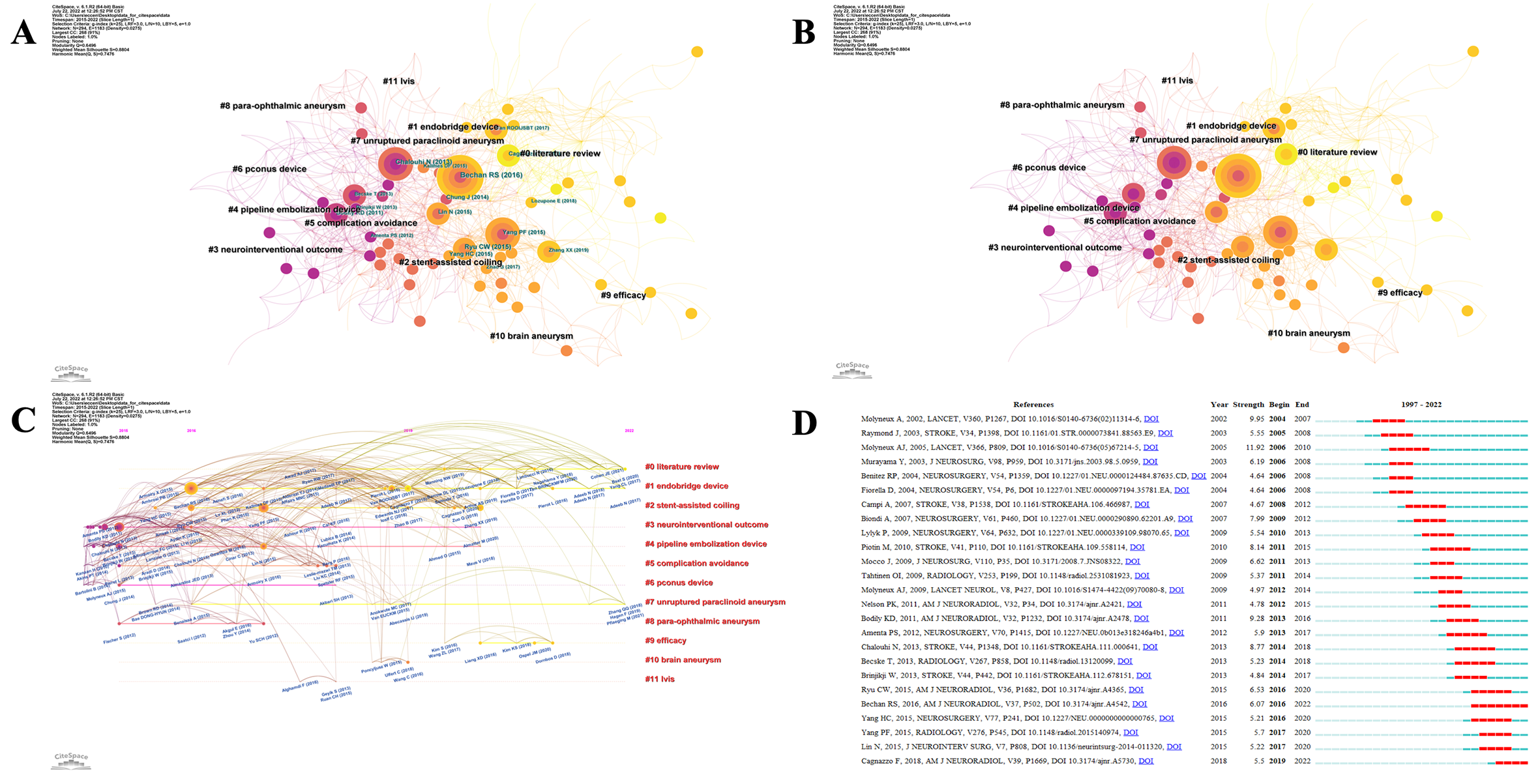

Supplement: Supplementary Figure 1 — (A) Co-citation reference network with cluster visualization from 2015 to 2022. (B) Visualization map of the corresponding clusters from 2015 to 2022. (C) Timeline visualization of co-citation references network. (D) Top 12 references with the strongest citation bursts. [file Image_1.TIFF]

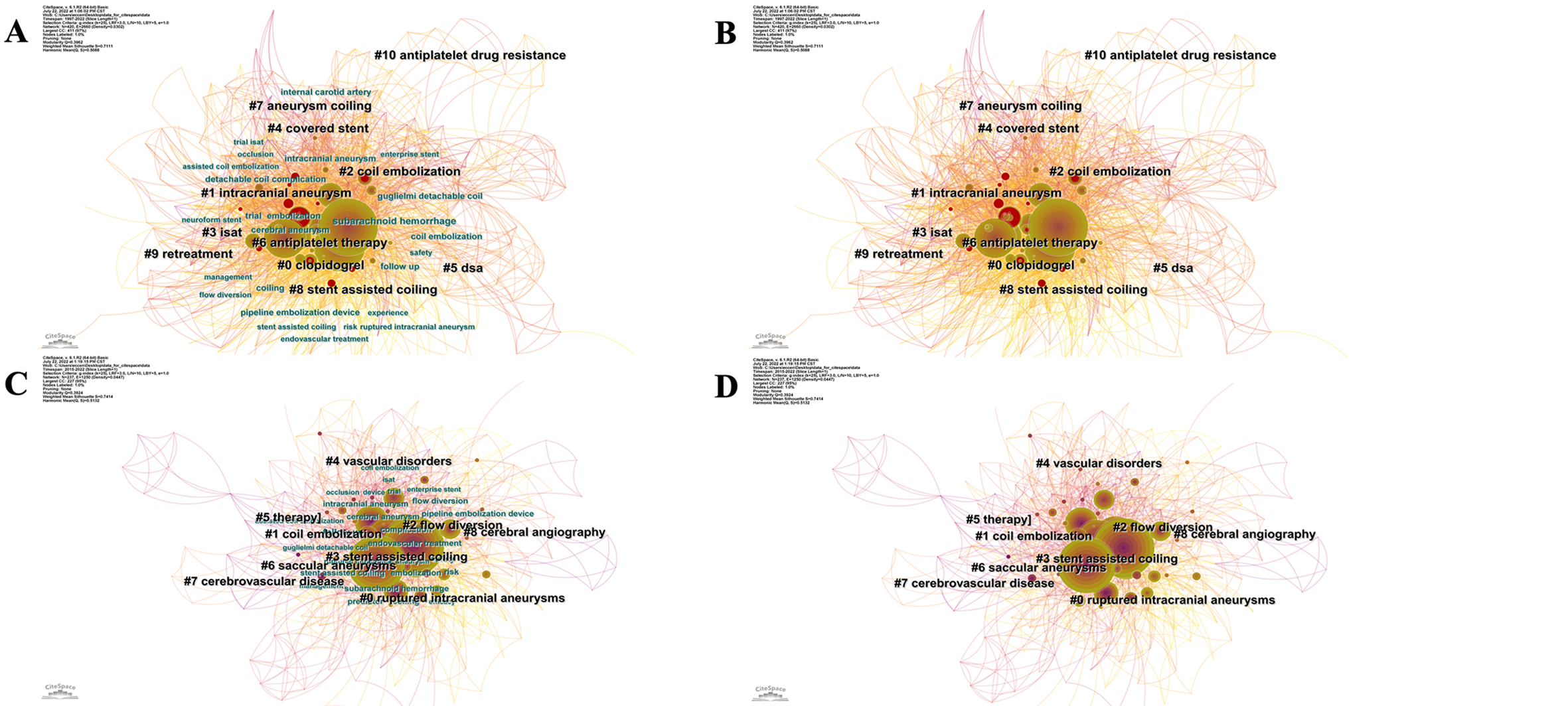

Supplement: Supplementary Figure 2 — Co-occurring author keyword networks with cluster visualization [(A) 1980–2021 and (C) 2015–2022]. Visualization map of the corresponding clusters [(B) 1980–2021 and (D) 2015–2022]. [file Image_2.TIFF]

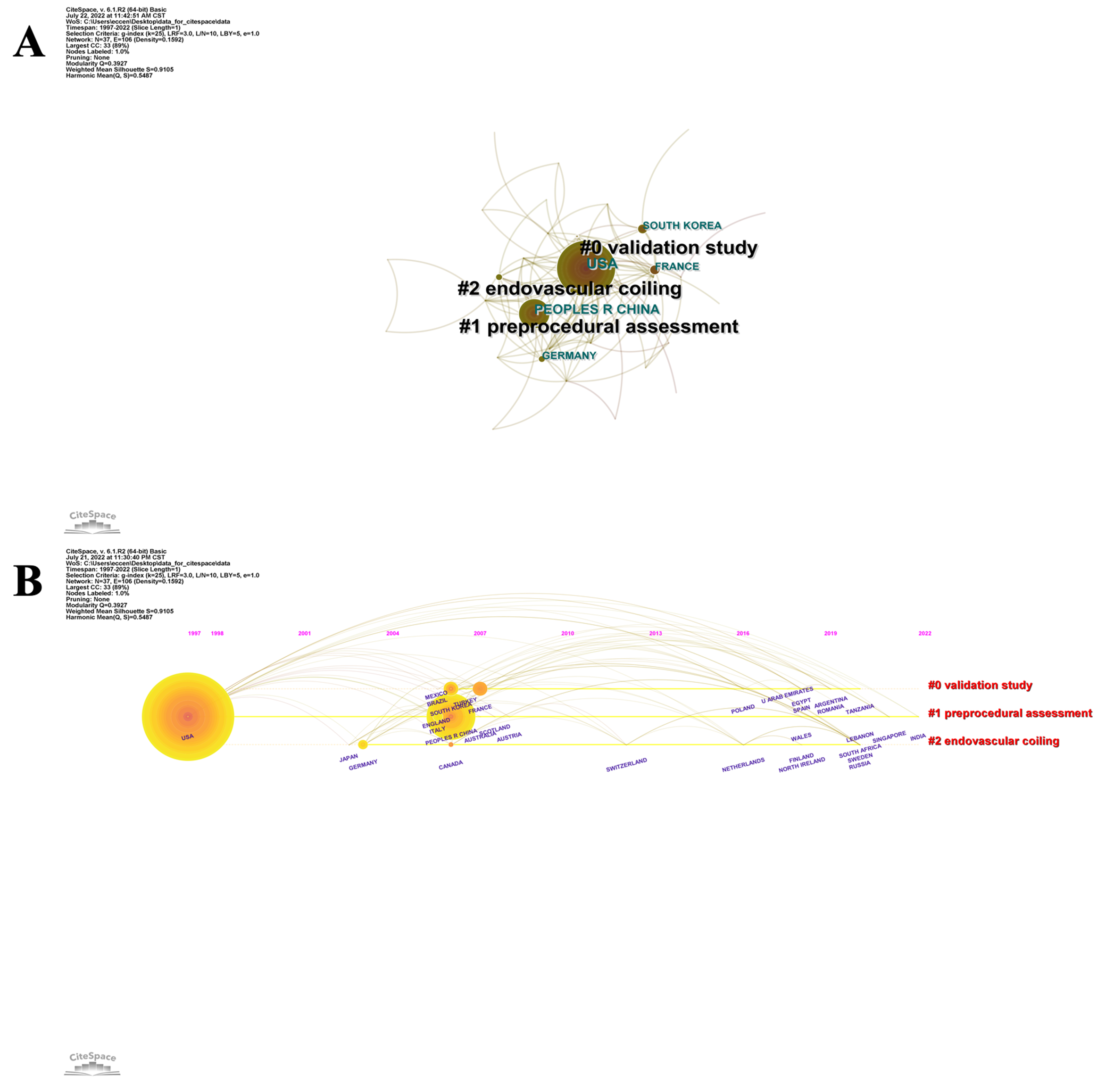

Supplement: Supplementary Figure 3 — (A) Country cooperation network with cluster visualization. (B) Timeline visualization of the country cooperation network. [file Image_3.TIFF]

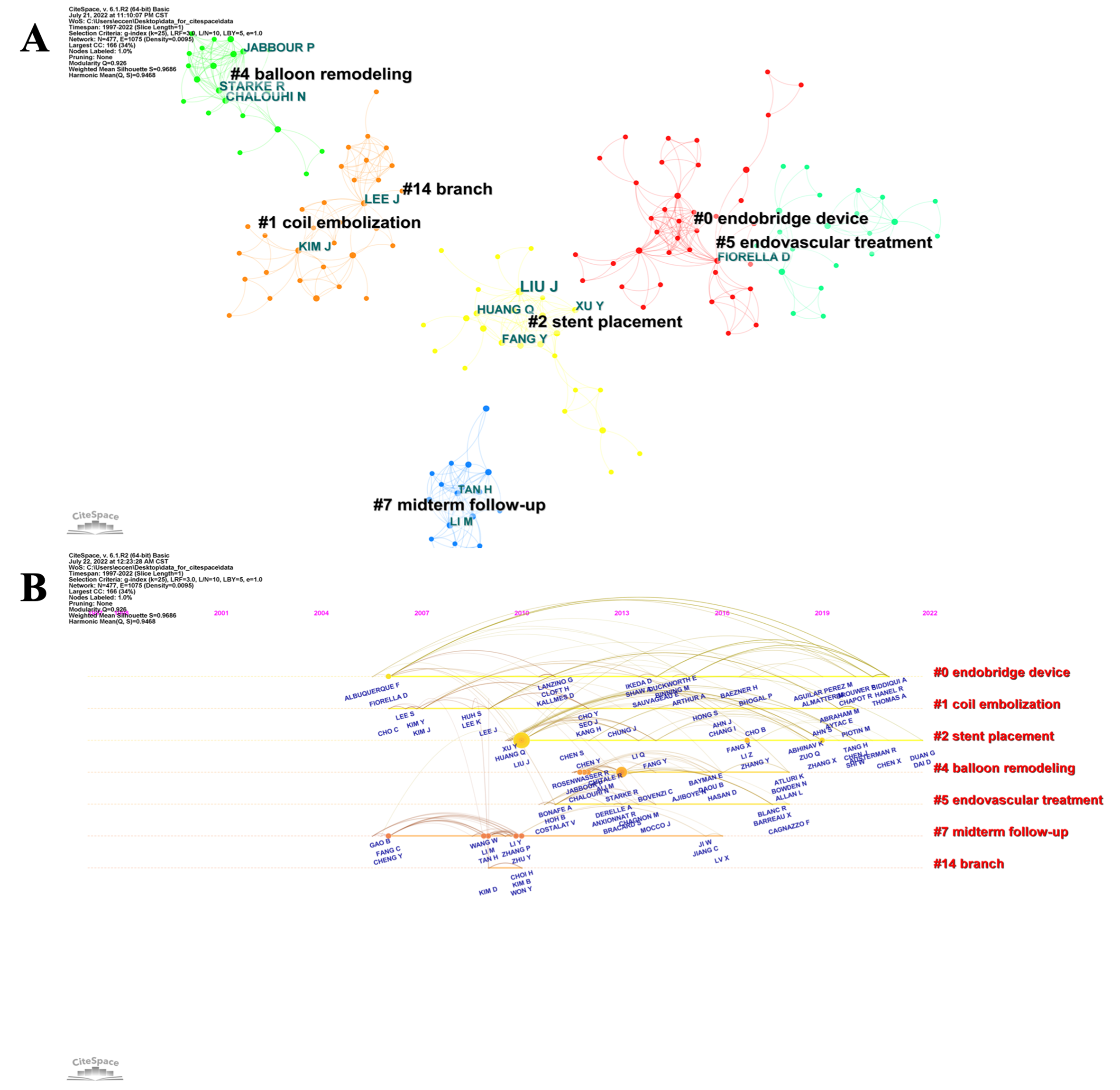

Supplement: Supplementary Figure 4 — (A) Author cooperation network with cluster visualization. (B) Timeline visualization of the author cooperation network. [file Image_4.TIFF]

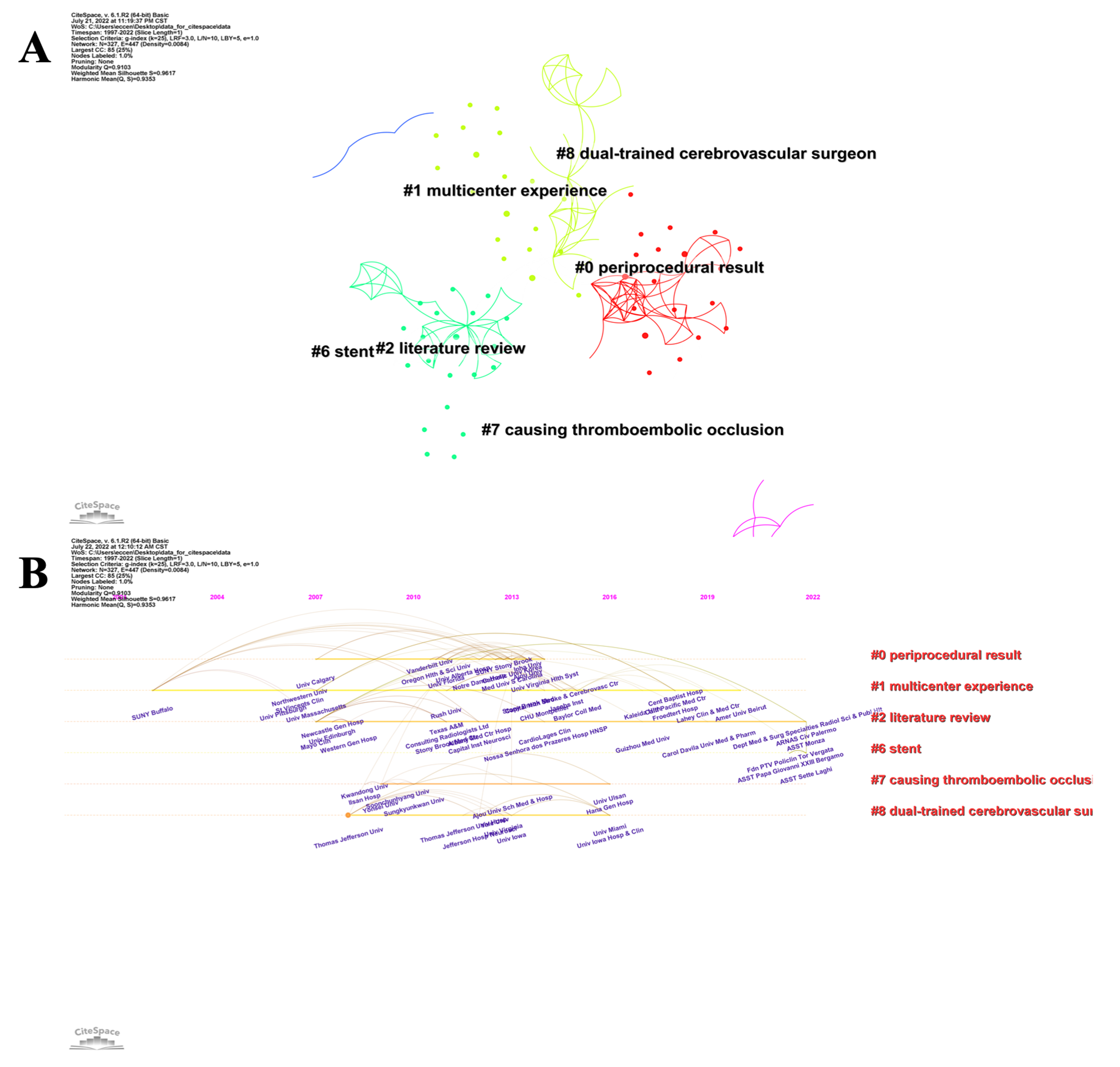

Supplement: Supplementary Figure 5 — (A) Institution cooperation network with cluster visualization. (B) Timeline visualization of the institution cooperation network. [file Image_5.TIFF]

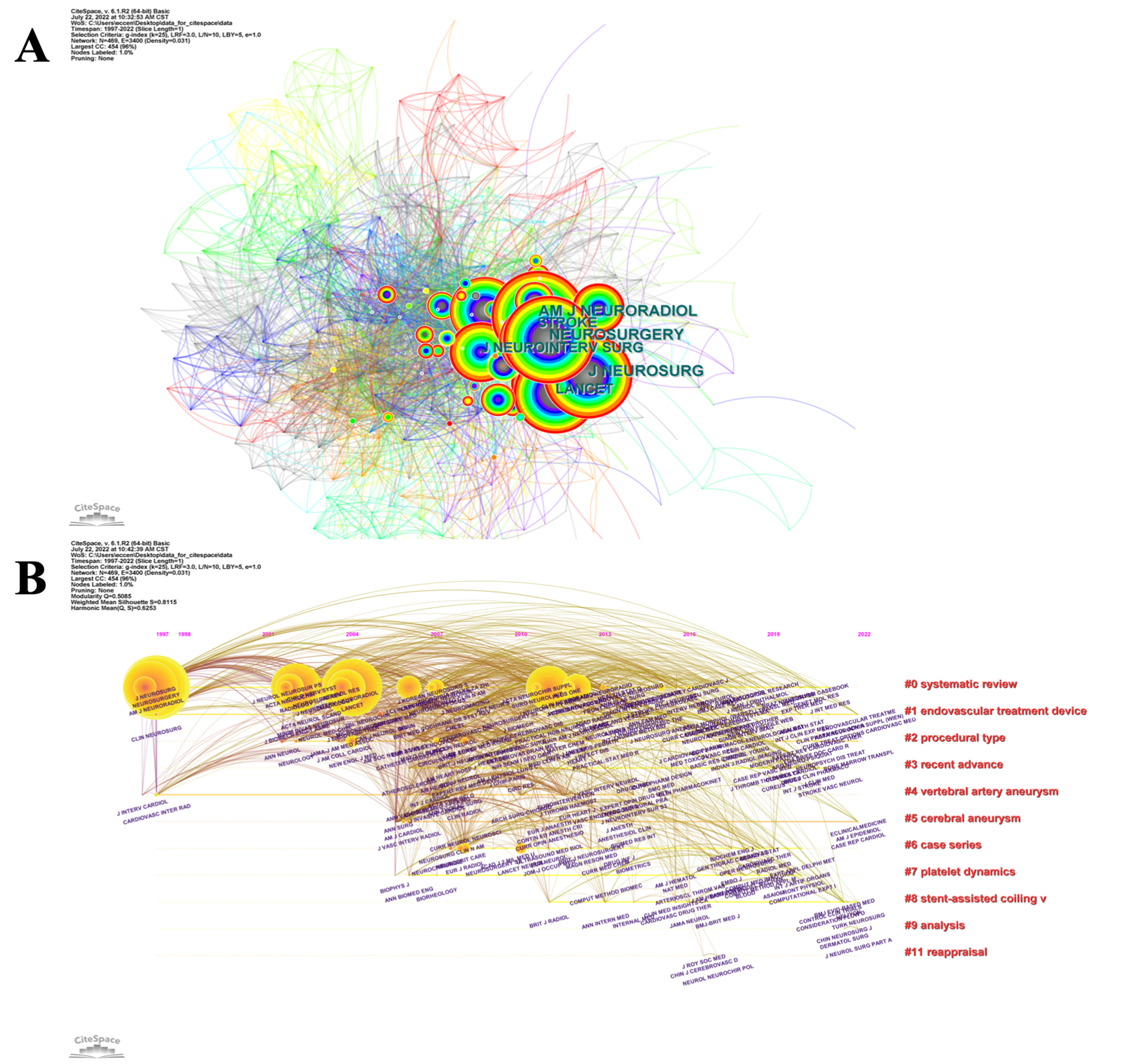

Supplement: Supplementary Figure 6 — (A) Co-cited journal network map. (B) Timeline visualization of the co-cited journals network map. [file Image_6.TIFF]
