# Supplementary material for: Worldwide productivity and research trends of publications concerning stent application in acutely ruptured intracranial aneurysms: A bibliometric study
Source: Front Neurol. 2022 Nov 11;13:1029613. doi: 10.3389/fneur.2022.1029613 (PMC9694826; doi:10.3389/fneur.2022.1029613)
Supplement: Supplementary file 7 [file Table_1.DOCX]

**Supplementary Table 1** The top ten countries with the most references

| Rank | Count | Centrity | year | Country/Region |
| --- | --- | --- | --- | --- |
| 1 | 102 | 0.87 | 1997 | USA |
| 2 | 57 | 0.1 | 2006 | PEOPLES R CHINA |
| 3 | 29 | 0.1 | 2006 | SOUTH KOREA |
| 4 | 22 | 0.05 | 2003 | GERMANY |
| 5 | 21 | 0.1 | 2007 | FRANCE |
| 6 | 15 | 0.01 | 2006 | ITALY |
| 7 | 14 | 0.04 | 2006 | CANADA |
| 8 | 12 | 0.01 | 2003 | JAPAN |
| 9 | 10 | 0.06 | 2006 | ENGLAND |
| 10 | 8 | 0 | 2008 | AUSTRIA |

**Supplementary Table 2** The top ten authors with the most references

| Rank | Count | Centrity | Year | Author |
| --- | --- | --- | --- | --- |
| 1 | 16 | 0.17 | 2010 | LIU J |
| 2 | 9 | 0.01 | 2012 | CHALOUHI N |
| 3 | 9 | 0.13 | 2009 | LEE J |
| 4 | 8 | 0.01 | 2013 | STARKE R |
| 5 | 7 | 0 | 2014 | FANG Y |
| 6 | 7 | 0.09 | 2006 | FIORELLA D |
| 7 | 7 | 0.03 | 2007 | KIM J |
| 8 | 7 | 0.01 | 2012 | JABBOUR P |
| 9 | 7 | 0 | 2010 | XU Y |
| 10 | 7 | 0 | 2010 | HUANG Q |

**Supplementary Table 3** The top ten institutions with the most references

| Rank | Count | Centrity | Year | Institution |
| --- | --- | --- | --- | --- |
| 1 | 9 | 0.07 | 2007 | Mayo Clin |
| 2 | 9 | 0.01 | 2008 | Thomas Jefferson Univ |
| 3 | 8 | 0 | 2006 | Shanghai Jiao Tong Univ |
| 4 | 8 | 0 | 2013 | Capital Med Univ |
| 5 | 7 | 0.01 | 2012 | Jefferson Hosp Neurosci |
| 6 | 6 | 0.09 | 2002 | SUNY Buffalo |
| 7 | 6 | 0 | 2007 | Dankook Univ |
| 8 | 5 | 0 | 2010 | Second Mil Med Univ |
| 9 | 5 | 0 | 2013 | Univ Iowa |
| 10 | 4 | 0 | 2014 | Hebei Med Univ |

**Supplementary Table 4** The top ten journals with the most references

| Year | 2018 | 2019 | 2020 | 2021 | 2022 |
| --- | --- | --- | --- | --- | --- |
| AMERICAN JOURNAL OF NEURORADIOLOGY | 21 | 23 | 24 | 26 | 26 |
| JOURNAL OF NEUROINTERVENTIONAL SURGERY | 17 | 23 | 24 | 25 | 25 |
| NEUROSURGERY | 20 | 20 | 22 | 24 | 24 |
| INTERVENTIONAL NEURORADIOLOGY | 13 | 14 | 18 | 19 | 21 |
| WORLD NEUROSURGERY | 5 | 12 | 14 | 16 | 16 |
| JOURNAL OF NEUROSURGERY | 10 | 12 | 13 | 13 | 14 |
| NEURORADIOLOGY | 7 | 7 | 7 | 7 | 7 |
| ACTA NEUROCHIRURGICA | 4 | 5 | 5 | 7 | 7 |
| CLINICAL NEUROLOGY AND NEUROSURGERY | 2 | 2 | 5 | 5 | 7 |
| CLINICAL NEURORADIOLOGY | 3 | 3 | 5 | 6 | 6 |

**Supplementary Table 5** The top ten journals with the most citations

| rank | frequent | degree | centrality | journal | halflife |
| --- | --- | --- | --- | --- | --- |
| 1 | 264 | 29 | 0.01 | AM J NEURORADIOL | 17.5 |
| 2 | 262 | 22 | 0 | NEUROSURGERY | 17.5 |
| 3 | 247 | 25 | 0 | J NEUROSURG | 17.5 |
| 4 | 238 | 23 | 0 | STROKE | 18.5 |
| 5 | 208 | 16 | 0 | LANCET | 10.5 |
| 6 | 163 | 22 | 0.01 | J NEUROINTERV SURG | 7.5 |
| 7 | 161 | 28 | 0.01 | NEURORADIOLOGY | 17.5 |
| 8 | 149 | 26 | 0.01 | RADIOLOGY | 11.5 |
| 9 | 121 | 19 | 0.02 | INTERV NEURORADIOL | 12.5 |
| 10 | 92 | 31 | 0.01 | WORLD NEUROSURG | 6.5 |
